# Second‐trimester multimetabolite panel for early preeclampsia rule‐out

**DOI:** 10.1002/ijgo.70983

**Published:** 2026-03-31

**Authors:** Matthews Silva Martins, Julyane N. S. Kaihara, Diego Fortes Salgueiro, Viviane Cunha Cardoso, Silvana Maria Quintana, Ricardo Carvalho Cavalli, Valerio Garrone Barauna, Valeria Cristina Sandrim

**Affiliations:** ^1^ Department of Physiological Sciences, Health Sciences Center Federal University of Espírito Santo Vitoria ES Brazil; ^2^ Department of Biophysics and Pharmacology, Institute of Biosciences of Botucatu São Paulo State University Botucatu SP Brazil; ^3^ School of Physical Education and Sport University of São Paulo (USP) Ribeirão Preto SP Brazil; ^4^ Department of Childcare and Pediatrics, Ribeirão Preto Medical School University of São Paulo Ribeirão Preto SP Brazil; ^5^ Department of Gynecology and Obstetrics, Ribeirão Preto Medical School University of São Paulo Ribeirão Preto SP Brazil

**Keywords:** metabolites, metabolomics, prediction, preeclampsia, rule‐out test

## Abstract

**Objective:**

We aimed to establish a high‐sensitivity, multimetabolite rule‐out model for the development of preeclampsia (PE), prioritizing minimizing false negatives to exclude low‐risk individuals from intensive surveillance confidently.

**Methods:**

In this prospective, nested case–control study, maternal serum samples were collected between 20^+0^ and 25^+6^ weeks of gestation, before the diagnosis of PE, from the BRISA (Brazilian Ribeirão Preto and São Luís) prenatal cohort. Participants were prospectively followed until delivery, and PE cases were diagnosed during follow‐up. Among 1400 enrolled women, 940 delivered at our institution; 30 were later diagnosed with PE, and 28 were included in the metabolomics analysis. The control group encompassed 28 healthy women, matched for maternal age and body mass index. A targeted metabolomics approach analyzed a 41‐metabolite panel using liquid chromatography coupled with tandem mass spectrometry. Eight metabolites below the detection limit in ≥15% of samples were excluded.

**Results:**

Compared to the control group, univariate analysis revealed lower methionine and glutamine concentrations and elevated threonine levels in the case group. Classification models based on the remaining 33 metabolites, using leave‐one‐out cross‐validation, achieved 100% sensitivity but had limited specificity (21%). A reduced four‐metabolite model maintained 100% sensitivity with 50% specificity, while our refined, best‐performing six‐metabolite model achieved 100% sensitivity and 61% specificity with a negative predictive value of 100%.

**Conclusion:**

Our models demonstrated considerable potential as diagnostic tools for ruling out PE, thereby reinforcing their future applicability in screening and monitoring strategies during gestation to improve clinical decision‐making and alleviate the burden on healthcare systems.

## 1 INTRODUCTION

Preeclampsia (PE) is a multisystem hypertensive disorder of pregnancy, defined by new‐onset hypertension after 20 weeks' gestation, accompanied by evidence of end‐organ dysfunction, such as proteinuria, impaired liver function, coagulopathy, pulmonary edema, new‐onset headache, or visual symptoms.^1^ PE remains one of the leading direct causes of maternal and perinatal mortality,^2^ with a global prevalence estimated between 2% to 8% of all pregnancies.^3^, ^4^ This condition has been associated with an increased risk of long‐term cardiovascular complications and an elevated susceptibility to chronic diseases in later life, even beyond the postpartum period.^4^, ^5^ Consequently, PE constitutes a major public health concern, yielding substantial repercussions for maternal and neonatal prognoses. However, the rapid progression and incompletely understood pathophysiology of PE limit the effectiveness of current preventive and therapeutic strategies.

This highlights an urgent clinical need for the screening of early and reliable predictive biomarkers for the detection of pregnant women at a high or low risk of developing PE. The identification of angiogenic imbalance as a central pathophysiological mechanism in PE has led to the development of predictive biomarkers, such as placental growth factor (PlGF) and the soluble fms‐like tyrosine kinase‐1 (sFlt‐1)/PlGF ratio.^6^, ^7^, ^8^ Other biomarkers include pregnancy‐associated plasma protein A (PAPP‐A2) and inhibin A,^9^ uric acid,^10^ asymmetric dimethylarginine (ADMA),^11^ homocysteine,^11^, ^12^ uterine artery Doppler indices,^12^ the microRNA miR‐204‐5p,^13^ and urinary markers such as albuminuria.^14^

Beyond these targeted markers, the expanding availability of “omic” approaches (e.g., genomics, proteomics, and metabolomics) has accelerated the discovery of novel PE‐related pathways and factors measurable in maternal blood,^15^, ^16^ making them suitable for biomarker investigation. Our group has shown that circulating metabolites and proteins might reflect the severity and underlying pathophysiology of hypertensive disorders of pregnancy.^17^, ^18^, ^19^, ^20^ Moreover, metabolomic studies have demonstrated potential for early prediction of PE.^21^, ^22^, ^23^, ^24^, ^25^ Notably, existing metabolomic studies were largely conducted during the first or early second trimester and mainly focused on single‐metabolite predictive models and/or overlooked rule‐out performance metrics.^22^, ^23^, ^24^, ^25^ This scenario underscores the importance of incorporating multivariate metabolomic panels to support rule‐out strategies, improve clinical decision‐making, and alleviate the burden on healthcare systems.

In this study, we leveraged the power of metabolomics during the second trimester of pregnancy, before PE diagnosis, in combination with multivariate modeling to address this need. Our primary objective was to identify a parsimonious panel of serum metabolites that could serve as a high‐sensitivity and high negative predictive value (NPV) rule‐out test for PE development, prioritizing minimizing false negatives to exclude low‐risk individuals from intensive surveillance confidently. A four‐metabolite combination (methionine, glutamine, aspartate, and pyruvate) achieved 100% sensitivity with 50% specificity, and our refined, best‐performing panel comprised six metabolites (aspartate, alanine, methionine, acetylcarnitine, tryptophan, and uric acid), reaching 100% sensitivity and 61% specificity with 100% NPV in our cohort.

## 2 MATERIALS AND METHODS

### 2.1 Study design and population

A prospective, nested case–control study was conducted within the BRISA (Brazilian Ribeirão Preto and São Luís) prenatal cohort, originally designed to investigate non‐classical risk factors associated with preterm birth and the impact of perinatal indicators on fetal and infant growth.^26^ The data reported here refer exclusively to a subset of the BRISA study, specifically the Ribeirão Preto cohort. Pregnant women were recruited during first‐trimester prenatal visits at ultrasound clinics and prenatal care offices and were invited to the Clinical Research Unit of the Ribeirão Preto Medical School, University of São Paulo (RPMS/USP), between January 2010 and June 2011. Maternal serum samples were collected prospectively during the second trimester (20^+0^ to 25^+6^ weeks of gestation), prior to the clinical diagnosis of PE. The participants were followed longitudinally until delivery, and PE cases were defined as women who subsequently developed PE during follow‐up. The present study was conducted in accordance with the Declaration of Helsinki and with the approval of the Research Ethics Committee of RPMS/USP, under protocol #4116/08, and all participants provided written informed consent.

**FIGURE 1:**
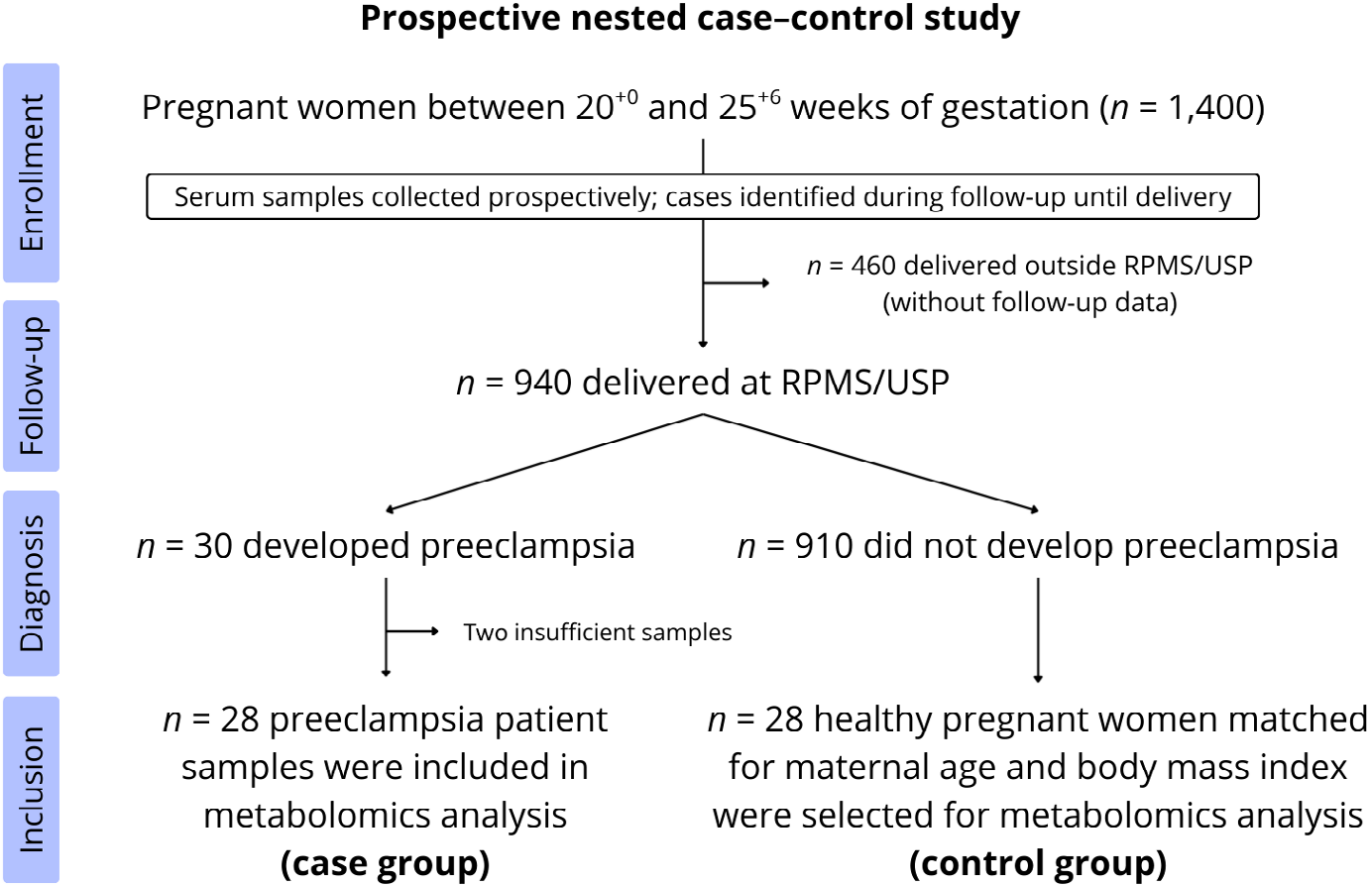
Participant flow diagram. Pregnant women were enrolled and maternal serum samples were collected prospectively during the second trimester, prior to clinical diagnosis of preeclampsia. Cases of preeclampsia were subsequently identified during longitudinal follow‐up until delivery.

Figure 1 provides an overview of the participant enrollment. A total of 1400 eligible pregnant women were invited to participate in the study, of whom 460 gave birth outside the RPMS/USP, whereas the remaining 940 pregnant women delivered at the study institution and had complete follow‐up data. Among these, 30 women (approximately 3.2%) developed PE. For the present metabolomics analysis, serum samples were available for 28 patients who developed PE (case group). These cases were individually matched to 28 healthy singleton pregnancies (control group) according to maternal age and body mass index at sampling. Eligible controls were restricted to participants who remained normotensive throughout pregnancy, had no diagnosis of hypertensive disorders, and did not develop other obstetric or clinical complications. From this eligible pool, control subjects were randomly selected, while ensuring comparable distributions of maternal age and body mass index at sampling. All controls originated from the same prospective cohort as cases, ensuring identical recruitment procedures, clinical follow‐up conditions, and biological sample collection, processing, and storage protocols. The sample size for the metabolomics analysis was determined by the number of incident PE cases occurring within the prospective cohort and the availability of stored serum samples.

### 2.2 Diagnostic criteria

The diagnosis of PE was made according to the criteria established by the American College of Obstetricians and Gynecologists^1^: new‐onset hypertension after 20 weeks of gestation (systolic blood pressure ≥140 mmHg and diastolic pressure ≥90 mmHg in two blood pressure measurements at rest, 4 h apart), and proteinuria (≥300 mg/L in 24‐h urine, protein/creatinine ratio ≥0.3, or a dipstick reading of 2+). In the absence of proteinuria, PE was diagnosed with new‐onset hypertension associated with thrombocytopenia, renal insufficiency, impaired liver function, pulmonary edema, and cerebral or visual symptoms. Further clinical, demographic, and obstetric data were obtained from the patient's medical records. Systolic and diastolic blood pressure and heart rate values at sampling and at the time of diagnosis of PE were obtained by the arithmetic means of at least two consecutive measurements. The formula for computing mean arterial pressure (MBP), using systolic and diastolic blood pressure (SBP and DBP), is presented in Equation (1).^27^ Pregnant women with multiple pregnancies, chronic hypertension, cancer, or autoimmune, renal, or hepatic diseases were not included in the case or control groups.

(1) $$\mathrm{MBP}=\frac{\mathrm{SBP}+2\mathrm{DBP}}{3}$$

### 2.3 Blood sampling

Maternal venous blood samples were collected by peripheral venipuncture in serum tubes with clot activator (BD Vacutainer, Becton‐Dickinson, São Paulo, Brazil). The tubes were rapidly centrifuged (1000 × g for 10 min) at room temperature, and the serum aliquots were stored at −80°C.

### 2.4 Metabolomics

Serum samples were transported on dry ice to IonMedicine Clinical Metabolomics (São Paulo, Brazil), where targeted metabolomic profiling was conducted using liquid chromatography coupled with triple quadrupole mass spectrometry detection (LC–MS/MS, Shimadzu 8060NX, Kyoto, Japan). The metabolite panel comprised 41 primary metabolites, carnitines, nucleosides, amino acids and detivatives; namely, cystine, aspartate, asparagine, serine, alanine, hydroxyproline, glutamate, glycine, glutamine, threonine, citrulline, proline, ornithine, lysine, histidine, arginine, gamma‐aminobutyric acid (GABA), trimethylamine N‐oxide, valine, carnitine, methionine, thymine, guanosine, cysteine, tyrosine, adenosine, ADMA, isoleucine, glutathione, leucine, phenylalanine, kynurenine, acetylcarnitine, tryptophan, taurine, malate, pyruvate, lactate, uric acid, succinate, and oxyglutathione.

A total of 200 μL of each sample was added to 10 μL of a derivatizing solution containing N‐ethylmaleimide (200 μM) and citric acid (2 mM) in liquid chromatography–mass spectrometry (LC–MS)‐grade methanol. The mixture was homogenized and incubated for 1 h at room temperature, followed by filtration through 0.22 μm syringe filters. Subsequently, an aliquot of 50 μL of the filtrate was transferred to a 96‐well plate and mixed with 200 μL of a protein‐precipitating solution (acetonitrile/isopropanol/formic acid, 69:30:1, v/v/v) containing stable isotopically labeled internal standards (Sigma‐Aldrich, St. Louis, MO, USA and Cambridge Isotope Laboratories, Tewksbury, MA, USA), followed by vortex agitation for 10 min and refrigeration at −20°C for 10 min. Samples were centrifuged at 1000 ×g for 10 min at 10°C, and the supernatants were transferred to a shallow‐well plate, diluted 1:1 with isopropanol/ultrapure water (3:7, v/v) for injection. Quality control procedures for the analytical runs included injections of three independent pooled samples. Metabolite profiling was conducted using liquid chromatography coupled with tandem mass spectrometry (LC–MS/MS) with polarity optimization in multiple reaction monitoring (MRM) mode. Separation was achieved on a Discovery HS F5 column (3 μm, 2.1 mm x 150 mm, Sulpeco, Sigma‐Aldrich) using a mobile phase consisting of water with 0.1% formic acid (A) and acetonitrile with 0.1% formic acid (B). The injection volume (5 μL) was performed using calibration curves generated from reference standards across multiple concentrations, and analyte identification was confirmed by specific MRM transitions and isotopically labeled internal standards. Data processing, including quantification of metabolite concentrations, chromatographic peak integration, and quality control assessments, was performed using LabSolutions CL (Shimadzu) and LabSolutions Insight softwares (Shimadzu). The mean coefficient of variation was 7.78% for the experiment. Further quality control metrics and parameters are presented in Table S1.

### 2.5 Univariate analysis

Statistical analyses were performed to assess differences in clinical, demographic, and obstetric data, as well as in metabolite levels between the case and control groups. First, data distribution was evaluated using the Shapiro–Wilk test. The unpaired *t*‐test, Welch's *t*‐test, and the Mann–Whitney test were used, as appropriate, to compare the groups. Categorical variables were compared using contingency tables and Fisher's exact test. These analyses were performed using GraphPad Prism version 9.5 (GraphPad Software, San Diego, CA, USA). A significant level of 5% was adopted for all comparisons.

For each group, the 95% confidence interval (CI) of the mean metabolite level was calculated using the *t*‐distribution. Additionally, the effect size for each comparison was estimated using Cohen's *d*, providing a standardized measure of the magnitude of the difference between groups. The results are summarized in a volcano plot, where the –log10 (*P*‐value) is plotted against the fold change (control/case, %), allowing visualization of both the magnitude of change and statistical significance.

A total of eight metabolites with concentrations below the detection limit in 15% or more of the samples were excluded from further analysis; namely, adenosine, GABA, guanosine, glutathione, hydroxyproline, malate, oxyglutathione, and thymine. The final dataset comprised 33 metabolites for subsequent analyses.

### 2.6 Score plots (PCA and PLS‐DA)

Visual discrimination between case and control groups based on all metabolites was assessed using principal component analysis (PCA) and partial least squares discriminant analysis (PLS‐DA) score plots. PCA is an unsupervised dimensionality reduction technique that extracts information from intercorrelated variables and represents large datasets in a multivariate space through score plots. Because it is unsupervised, PCA does not rely on predefined class labels, and the natural patterns and clusters observed are inherent to the data.^28^, ^29^ PLS‐DA also performs dimensionality reduction; however, as a supervised technique, it incorporates class‐label information into the analysis, thereby maximizing covariance with the predictors. The results are likewise represented in a new multivariate space through a score plot, optimized for class separation.^30^ All score plots were generated with class ellipses representing the 95% confidence interval.

### 2.7 Multivariate classification analysis

To construct the prediction model for ruling out PE, PLS‐DA was used as the classification algorithm, with model performance evaluated using a leave‐one‐out cross‐validation (LOOCV) strategy.^31^ In this approach, the dataset is iteratively partitioned into training and testing subsets. In each iteration, *n* – 1 samples are used to build the model, while the remaining sample is used to test its predictive ability by determining the correct class label. This process is repeated until all samples have been tested, after which a final confusion matrix is generated, summarizing correct and incorrect classifications. From this, diagnostic metrics, including sensitivity, specificity, accuracy, NPV, and positive predictive value (PPV), are derived.

By definition, a rule‐out test must exhibit high sensitivity. Sensitivity is defined as the probability of a positive result among diseased individuals, calculated as the ratio of true positives to the sum of true positives and false negatives. Consequently, a highly sensitive test minimizes the number of false negatives, making a negative result more reliable for ruling out the disease and excluding patients who will not develop PE during pregnancy.^32^, ^33^ Sensitivity was optimized by analyzing the receiver operating characteristic (ROC) curve. The ROC curve plots the true positive rate (sensitivity) against the false positive rate (1 – specificity) across all possible thresholds, illustrating the trade‐off between correctly identifying positive and negative cases. Subsequently, the threshold that maximizes the sensitivity was applied to classify the samples.^34^ Additionally, NPV and PPV were calculated to indicate the probability that a positive or negative test result correctly reflects the true disease status (presence or absence), respectively.

Subsequently, a model was first constructed using 33 metabolites, and additional models were then built with reduced subsets. To identify the minimal number of metabolites required to produce the most accurate rule‐out test, an algorithm was systematically implemented to evaluate all possible combinations of two to six metabolites.

In parallel, variable importance in projection (VIP) scores were calculated for each model to assess each metabolite's relative contribution to classification. The VIP score reflects the weighted influence of each predictor on the model's latent structure, accounting for both the strength of association with the outcome and the amount of variation explained by the component.

### 2.8 Optimization of latent variables

For each evaluated combination of metabolites, a crucial step was optimizing the number of latent variables (LVs), a key hyperparameter in the PLS‐DA model that controls its complexity and prevents overfitting. The optimal number of LVs was selected using a grid search procedure. For each specific set of metabolites, multiple PLS‐DA models were constructed, varying the number of LVs across a predefined range (e.g., 1 to 5). The performance of each LV configuration was rigorously evaluated using a full LOOCV strategy. In this process, for a fixed number of LVs (e.g., two LVs), the model was trained and validated on the entire dataset via LOOCV. The out‐of‐sample predictions from all iterations were aggregated, and the overall performance was quantified by calculating the area under the ROC curve (AUC). This procedure was repeated for each LV value in the search range. Finally, the number of LVs yielding the maximum AUC was selected as the optimal choice for that specific metabolite combination. All subsequent diagnostic metrics for that model, including sensitivity and specificity, were then derived based on this optimal LV configuration.

### 2.9 Permutation analysis

To assess the statistical reliability of the rule‐out model, a permutation test was performed. In this approach, the true class labels of the patients are randomly shuffled to generate permuted datasets with no real class separation. A total of 500 permuted models were constructed, each with 40–60% of the class labels randomly reassigned. The distribution of sensitivities obtained from these permuted models was then compared with the sensitivity of the original model. The reliability of the model is confirmed when its sensitivity falls outside the range of the permuted distribution. In addition, a ROC curve of the original model was plotted alongside the mean ROC curve of the permuted models with ± standard deviation.^35^

### 2.10 Data preprocessing

All analyses were conducted in the Scientific Python Development Environment (Spyder 6.0.3) running Python 3.12.3. The libraries employed included scikit‐learn (1.6.1), NumPy (2.2.0), pandas (2.2.3), and Matplotlib (3.10.0). For multivariate analysis, the data were preprocessed with the StandardScaler function, which removes the mean and scales the features to unit variance.

## 3 RESULTS

### 3.1 Demographic and clinical characteristics of the cohort at sampling and preeclampsia diagnosis

The clinical, demographic, and obstetric characteristics of the control and case groups are presented in Table 1. No statistically significant differences were observed between the groups regarding maternal age or the distribution of White women, primiparas, and smokers. The case group exhibited higher systolic, diastolic, and mean blood pressure levels at the time of serum sample collection compared to the control group, although all blood pressure values remained below the diagnostic threshold for gestational hypertension or PE.^1^ Heart rate, body mass index, and gestational week at sampling did not differ significantly between the two groups. The deliveries in the case group occurred earlier and resulted in newborns with lower weight and height compared to the control group. Despite this, newborns in both groups exhibited equivalent Apgar scores at 1 and 5 min. Table S2 presents a summary of the clinical data and biochemical test results of pregnant women in the case group at the time of PE diagnosis.

**TABLE 1:** Clinical, demographic, and obstetric characteristics of the study subjects at sampling.

Parameter	Control	Case	*p*‐value
Maternal age (years)	28.0 ± 1.1	28.0 ± 1.1	0.94
Ethinicity (% White)	11 (39.3)	16 (57.1)	0.28
Primiparity (%)	11 (39.3)	14 (50.0)	0.59
Smoking (%)	2 (7.1)	4 (14.3)	0.67
SBP (mmHg)	109.5 ± 1.7	117.5 ± 2.2^a^	<0.01
DBP (mmHg)	66.6 ± 1.0	74.1 ± 1.7^a^	<0.01
MBP (mmHg)	80.9 ± 1.1	88.6 ± 1.8^a^	<0.01
Heart rate (bpm)	82.2 ± 2.1	78.9 ± 1.9	0.25
BMI (kg/m^2^)	30.9 ± 0.8	30.7 ± 1.1	0.92
GAS (weeks)	23.0 [22.0–24.0]	24.0 [22.0–24.0]	0.85
GAD (weeks)	39.0 [38.0–40.0]	38.5 [36.3–39.8]^a^	<0.01
Newborn weight (g)	3406.0 ± 100.8	2849.0 ± 193.1^a^	0.01
Newborn length (cm)	50.0 [48.6–51.0]	49.0 [46.8–50.5]^a^	0.02
APGAR Score 1 <7 (%)	5 (17.9)	8 (28.6)	0.53
APGAR Score 2 <7 (%)	0 (0.0)	2 (7.1)	0.49

*Note*: Data are presented as mean ± standard error of mean, median [first quartile – third quartile], or absolute numbers (frequency in percentage).

Abbreviations: BMI, body mass index; DBP, diastolic blood pressure; GAD, gestational age at delivery; GAS, gestational age at sampling; MBP, mean blood pressure; SBP, systolic blood pressure.

^a^ *p* <0.05 versus control.

### 3.2 Methionine, glutamine, and threonine concentrations differ between case and control groups

**FIGURE 2:**
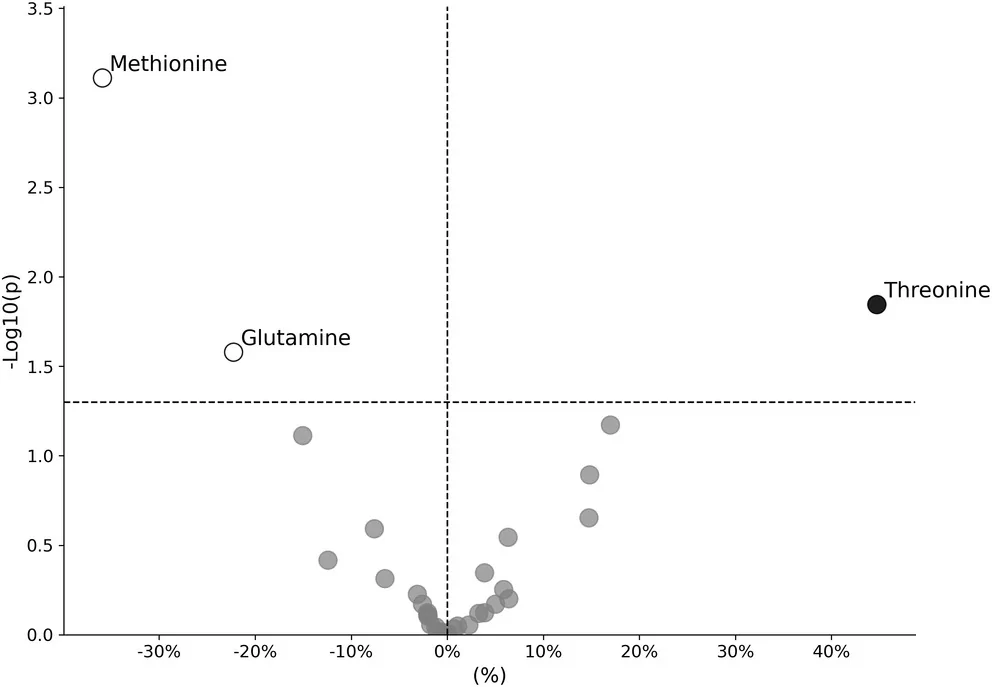
Volcano plot of serum metabolite changes in control women compared to those who developed preeclampsia. Black points represent upregulated metabolites, and white points represent downregulated metabolites in the case group.

Univariate analysis revealed significant alterations in the levels of three serum metabolites in patients who developed PE compared to the control group (Figure 2). Methionine levels were significantly lower in the case group (mean: 0.76 μmol/L; 95% CI: 0.60–0.91) compared to controls (1.18 μmol/L; 95% CI: 0.99–1.37; *P* = 0.0008, Cohen's *d* = −0.95), corresponding to a 35% reduction. Similarly, glutamine levels were reduced by 22% in cases (13.63 μmol/L; 95% CI: 11.67–15.58) relative to controls (17.53 μmol/L; 95% CI: 14.64–20.42; *P* = 0.026, *d* = −0.61). In contrast, threonine levels were significantly higher among cases (5.05 μmol/L; 95% CI: 4.03–6.06) than in controls (3.49 μmol/L; 95% CI: 2.74–4.23; *P* = 0.014, *d* = +0.68), representing a 44% increase. The concentrations of the 30 metabolites that were similar between groups are provided in Table S3.

### 3.3 Six‐metabolite panel achieves 100% sensitivity and negative predictive value for ruling out preeclampsia

**FIGURE 3:**
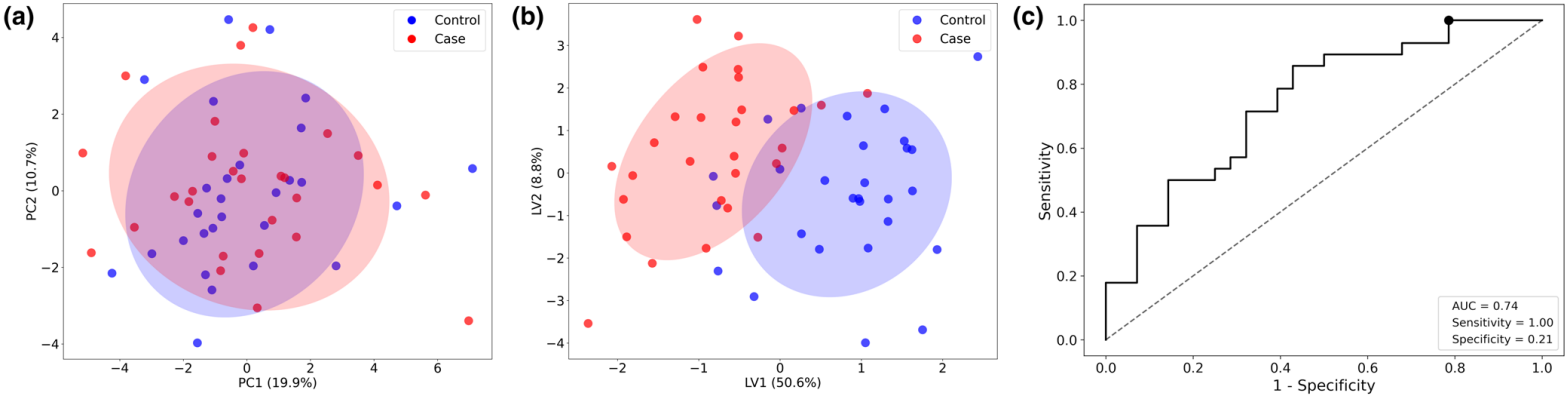
Multivariate analyses and classification performance of serum metabolomic profiles for women who develop preeclampsia. (a) Principal component analysis (PCA) and (b) partial least squares discriminant analysis (PLS‐DA) score plots using all 33 metabolites, and (c) ROC curve showing the model metric performance.

When evaluating the complete metabolomic profile (multivariate analysis of the 33 metabolites), an unsupervised PCA failed to clearly discriminate between the groups (Figure 3a; PC1 explained approximately 20% of data variance and PC2 approximately 11%). However, a supervised PLS‐DA demonstrated a clear separation between PE cases and controls (Figure 3b). An initial classification model using all 33 metabolites achieved the desired 100% sensitivity but with a low specificity of 21% (AUC = 0.74), limiting its clinical application as a rule‐out test (Figure 3c).

**FIGURE 4:**
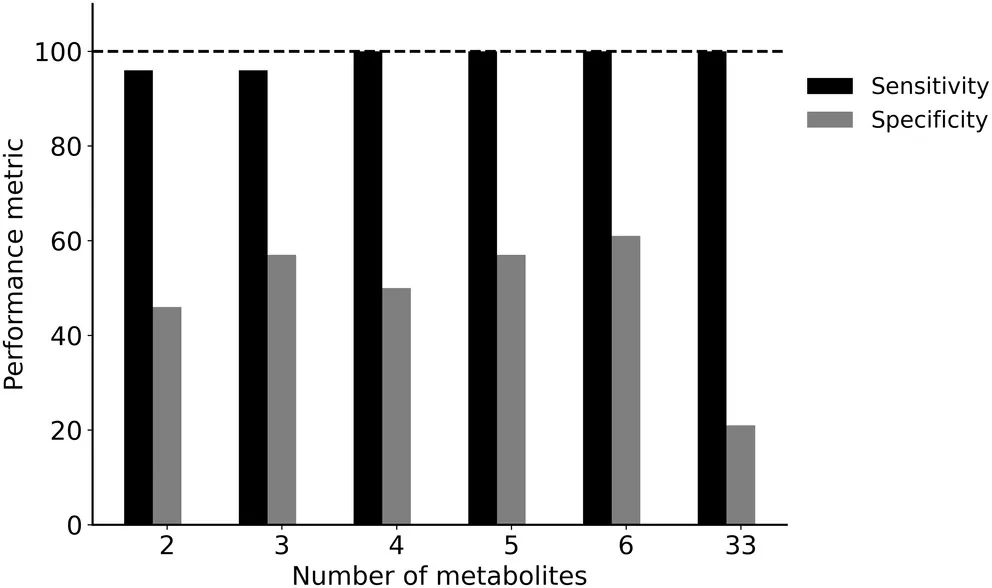
Sensitivity and specificity of PLS‐DA models constructed using subsets of two to six metabolites and the full panel (33 metabolites).

Next, we attempted to develop a predictive model with a reduced metabolite panel. To optimize specificity and create a smaller panel, we systematically investigated the minimum number of metabolites required to maintain 100% sensitivity. As illustrated in Figure 4, models with combinations of four, five, and six metabolites achieved this goal. The complete list of every possible combination of two to six metabolites and their metrics is presented in Tables S4–S8. It can be observed that none of the models with the combination of two or three metabolites achieved 100% sensitivity, which was only observed in models with four, five, and six metabolites. Among these, the analysis revealed that the four‐metabolite panel (methionine, glutamine, aspartate, and pyruvate) constitutes the minimum set necessary to achieve 100% sensitivity (with 50% specificity), and the model including six metabolites demonstrated slightly better performance, achieving 100% sensitivity and 61% specificity.

Table 2 presents the complete set of diagnostic metrics for the best‐performing model using combinations of two to six metabolites. Notably, the model with six metabolites (aspartate, alanine, methionine, acetylcarnitine, tryptophan, and uric acid) reached an accuracy of 80% and an NPV of 100%, a particularly relevant metric for the assessment of rule‐out tests. Remarkably, methionine was a constant component in all top‐performing models (from two to six metabolites) and consistently presented the highest VIP score.

**TABLE 2:** Performance metrics and VIP scores of the best models among the combinations of 2 to 6 metabolites and the model with all 33 metabolites.

Number of metabolites	Metabolites (VIP score)	Accuracy	Sensitivity	Specificity	NPV	PPV
2	Methionine (1.24), acetylcarnitine (0.68)	71	96	46	99	8
3	Methionine (1.51), acetylcarnitine (0.83), Isoleucine (0.13)	77	96	57	99	11
4	Methionine (1.46), glutamine (0.98), aspartate (0.85), pyruvate (0.41)	75	100	50	100	9
5	Methionine (1.59), aspartate (0.91), acetylcarnitine (0.89), uric acid (0.79), tryptophan (0.50)	79	100	57	100	11
6	Methionine (1.71), aspartate (0.96), acetylcarnitine (0.95), uric acid (0.87), tryptophan (0.51), alanine (0.47)	80	100	61	100	12
33	All	61	100	21	100	6

*Note*: Accuracy, sensitivity, specificity, NPV, and PPV are presented as percentages.

Abbreviations: NPV, negative predictive value; PPV, positive predictive value; VIP, variable importance in projection.

**FIGURE 5:**
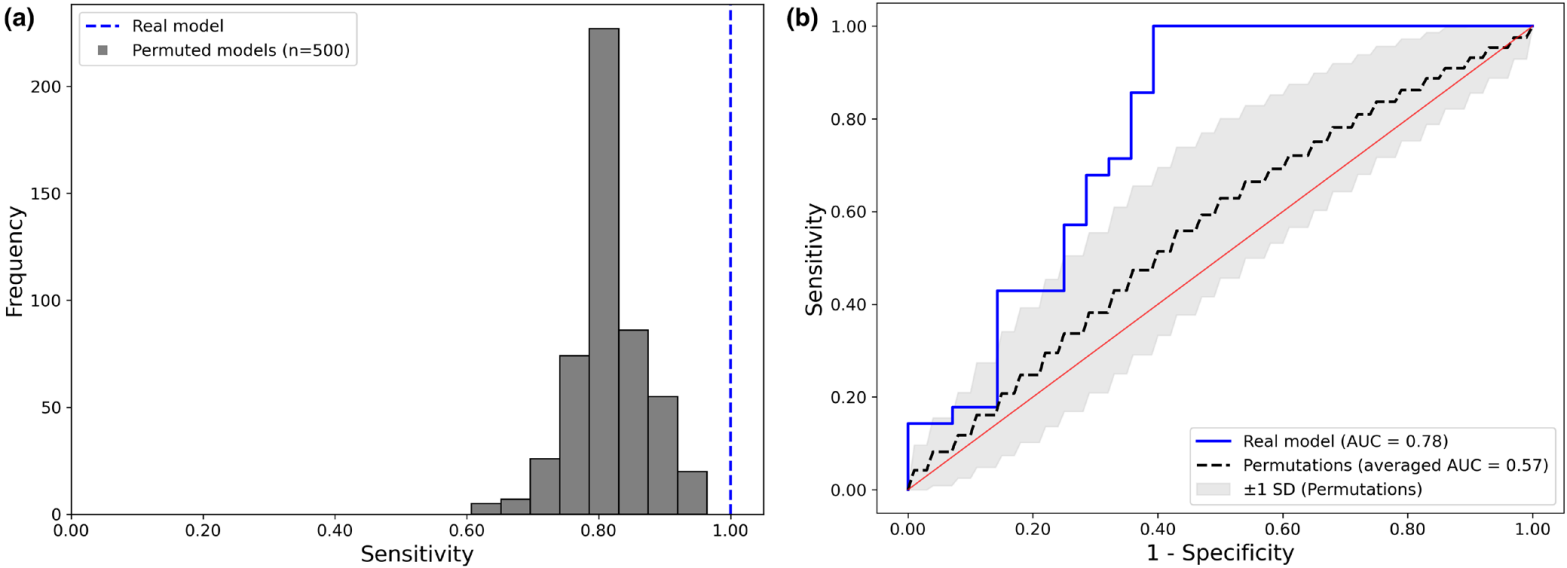
Permutation testing of the partial least squares discriminant analysis (PLS‐DA) model. (a) Distribution of sensitivity values obtained from 500 partially permuted models (gray bars), compared with the real model (dashed blue line). (b) Receiver operating characteristic (ROC) curve of the real model (blue) versus the average ROC of the permuted models (dashed black line, shaded area represents ± standard definition).

To evaluate the robustness of the six‐metabolite model, a permutation test was performed, and the results are presented in Figure 5. In Figure 5a, the distribution of sensitivity values from the 500 permuted models is displayed as gray bars, while a dashed blue line represents the sensitivity of the original model. The sensitivity of the original model lies completely outside the distribution of the permuted models, confirming that its performance was not achieved by chance. Figure 5b shows a comparison between the ROC curve of the original model and the average ROC curve of the permuted models. The AUC of the original model (blue line, 0.78) was substantially higher than the average of the 500 permuted models (dashed black line, 0.57).

## 4 DISCUSSION

In this prospective study, we aimed to develop a metabolomic rule‐out test for PE using serum samples collected between 20^+0^ and 25^+6^ weeks of gestation. While a full 33‐metabolite panel achieved the 100% sensitivity target, its specificity was clinically unviable at 21%. Through a systematic analysis of all possible smaller combinations, a four‐metabolite panel (methionine, glutamine, aspartate, and pyruvate) achieved 100% sensitivity and 50% specificity, and we identified an optimal six‐metabolite panel (aspartate, alanine, methionine, acetylcarnitine, tryptophan, and uric acid) that maintained 100% sensitivity while significantly improving specificity to 61% with 100% NPV. The robustness of this model was confirmed by permutation testing, highlighting its potential for future validation as a clinical tool.

From a biochemical perspective, the metabolites that constitute our predictive panels represent related disturbances of the one‐carbon and mitochondrial energy metabolisms, both of which are crucial for endothelial homeostasis. Consistent with our data, diminished levels of methionine have been previously identified during the first half of PE pregnancies,^36^, ^37^, ^38^ and such reductions might indicate impaired transmethylation flux through the S‐adenosylmethionine/S‐adenosylhomocysteine cycle. This disruption can have implications for DNA and histone methylation patterns and consequently affects the expression of angiogenesis and endothelial function mediators, such as endothelial nitric oxide synthase, vascular endothelial growth factor, placental growth factor, and tissue inhibitor of metalloproteinase‐3.^39^, ^40^, ^41^, ^42^, ^43^ Disturbances in angiogenesis and endothelial function have been identified as being pivotal to the pathophysiology of PE.^4^

Parallel changes in aspartate and alanine indicate impaired anaplerotic flux into the tricarboxylic acid cycle and redox imbalance (expressed as the ratio of nicotinamide adenine dinucleotide to its reduced form, reduced nicotinamide adenine dinucleotide), which is in line with the mitochondrial dysfunction reported in PE placentas.^44^, ^45^, ^46^ Moreover, glucogenic amino acids alanine and glutamine are substrates for their conversion to pyruvate in gluconeogenesis, ultimately linked to the liver–alpha‐cell axis, glucagon secretion, insulin resistance, hyperaminoacidemia, and mitochondrial dysfunction.^47^, ^48^ The increased inflammatory demand and oxidative stress that are hallmarks of PE are consistent with the decrease in circulating glutamine levels that we observed before the clinical onset of symptoms. Because glutamine is an important source for activated immune pathways,^49^, ^50^ lower glutamine concentrations might indicate an augmented glutamine uptake by immune cells during subclinical alterations preceding PE. Kenny and collaborators reported a similar reduction in circulating glutamine at 15 weeks in pregnancies that later developed PE,^37^ whereas Bahado‐Singh and colleagues observed elevated glutamine concentrations earlier in gestation (11–13 weeks).^36^

Alterations in acetylcarnitine provide additional evidence of disrupted mitochondrial β‐oxidation, accumulation of acetyl‐CoA, and fatty acid translocation, consistent with elevated lipid peroxidation and oxidative stress.^46^, ^51^ Finally, changes in uric acid and tryptophan are consistent, respectively, with a nucleotide‐binding oligomerization domain‐, leucine‐rich repeat‐, and pyrin domain‐containing protein 3‐dependent inflammasome activation and an influence of oxidative damage on immune function, with the latter perhaps representing an indoleamine 2,3‐dioxygenase‐driven metabolism of tryptophan down the kynurenine pathway with consequent maternal–fetal immunotolerance.^52^

Compared with previous metabolomic studies that relied mainly on single‐metabolite associations and/or univariate statistical models, this study demonstrates clinical benefit from a multivariate modeling approach specifically designed to maximize rule‐out performance. Instead of just differential metabolites, our study emphasizes the synergistic contribution of multiple biochemical pathways that collectively enhance predictive sensitivity. This methodological shift from isolated biomarker discovery toward integrative, high‐sensitivity exclusion modeling highlights a more clinically actionable paradigm for metabolomics in PE screening. Moreover, by restricting our analyses to a narrower interval within the second trimester, we potentially minimize physiological gestational variability related to pregnancy progression. In the context of the existing knowledge, our study advances the field by introducing a multimetabolite panel with relevant clinical utility as a rule‐out test, achieving high sensitivity and high NPV. A test with 100% sensitivity and a corresponding 100% NPV ensures that a negative result reliably excludes the disease, providing a trustworthy outcome for clinical decision‐making.^53^ Importantly, the metabolites included in our model were measured weeks before the potential clinical diagnosis, thereby reinforcing the test's efficacy in facilitating effective clinical triage and accurately identifying women who are unlikely to develop PE later in pregnancy.

To illustrate the clinical impact of this six‐metabolite test, if applied to a hypothetical cohort of 1000 pregnant women with a 3% disease prevalence, it would correctly rule out PE in 592 of the 970 unaffected individuals, and no affected patient would be missed. This represents the confident exclusion of nearly 59% of the total cohort from high‐risk surveillance, markedly reducing the burden of follow‐up with unnecessary monitoring and associated healthcare costs. Consequently, clinical resources and preventive interventions could be more efficiently directed toward the remaining truly higher‐risk population, ultimately improving the overall effectiveness of clinical management.

A particularly noteworthy finding of our study is the discrepancy between the univariate and multivariate analyses. Biological systems inherently exhibit high‐dimensional interdependence among variables, and the choice of analytical framework directly influences interpretability and robustness. Choosing between univariate and multivariate analyses can significantly affect the interpretation, precision, and validity of results. Univariate analysis, although simple and useful for preliminary screening, examines variables in isolation and, therefore, neglects potential correlation or confounding effects inherent to complex biological networks. Multivariate analysis involves the simultaneous analysis of multiple outcome variables, accounting for their correlations. This approach is essential when outcomes are related, as it provides more robust and precise inferences.^54^ Metabolites that were individually significant in the univariate analysis, such as threonine and glutamine, were ultimately excluded from our top‐performing six‐metabolite model. Conversely, key components of our final panel (aspartate, acetylcarnitine, uric acid, tryptophan, and alanine) would not have been prioritized based solely on univariate statistics. This difference highlights a fundamental strength of multivariate modeling: while univariate analysis assesses variables in isolation, a multivariate approach considers the covariance structure within the data. A predictive signature often relies on the collective, subtle contributions of multiple variables, whose predictive power only becomes apparent when analyzed in combination. This phenomenon is well documented in biomarker discovery, where the most robust models are frequently composed of variables that are not individually the most statistically significant. Indeed, the ability to perform exhaustive combinatorial analysis, as was done in this study, and to uncover these intricate patterns is a direct result of recent advancements in computational power and the application of machine learning tools.

For completeness, we note that the six‐metabolite panel reported in the main text was not the only configuration to satisfy the prespecified rule‐out criterion. In the exhaustive combinatorial search, multiple sets achieved 100% sensitivity: three of 45 760 four‐metabolite panels (approximately 0.0066%), 91 of 261 626 five‐metabolite panels (approximately 0.0348%), and 494 of 1 107 568 six‐metabolite panels (approximately 0.0446%). However, all these alternatives had lower specificity than the panel presented. Accordingly, we report in the manuscript the solution with the highest specificity among those meeting 100% sensitivity and 100% NPV, while providing a complete enumeration of candidate panels in the supplementary material to ensure transparency and reproducibility.

Although our cohort was not controlled by socioeconomic, dietary, environmental, or genetic factors that might influence metabolomic profiles, this limitation is partially mitigated by rigorous patient matching and standardized sample collection protocols. Moreover, the use of well‐characterized samples collected prospectively, before the clinical onset of PE and within a narrowly defined gestational window strengthens the temporal association between early metabolomic alterations and subsequent disease development while minimizing confounding effects related to gestational age. Collectively, these methodological strengths support the robustness of the proposed multimetabolite panel as a rule‐out tool. Further validation in independent cohorts and longitudinal cohorts will be crucial to confirm its clinical utility and to advance the integration of metabolomics into PE screening protocols. Additionally, future studies must include cost‐effectiveness analyses on a broader scale to determine the economic viability and to justify its integration into public health policies for PE.

## 5 CONCLUSION

Our analysis identified a multivariate model of six circulating metabolites that distinguishes pregnant women who will not subsequently develop PE. This model demonstrated considerable potential as a diagnostic tool for ruling out the possibility of developing the hypertensive disorder, thereby reinforcing its future applicability in improving screening and monitoring strategies during gestation.

## AUTHOR CONTRIBUTIONS

**Matthews Silva Martins:** Writing—Original Draft, Writing—Review & Editing, Conceptualization, Data Curation, Software, and Formal Analysis. **Julyane N. S. Kaihara:** Writing—Review & Editing, Conceptualization, Data Curation, Formal Analysis, Visualization, Methodology, and Funding Acquisition. **Diego Fortes Salgueiro:** Conceptualization, Writing—Review & Editing. **Viviane Cunha Cardoso:** Resources, Writing—Review & Editing. **Silvana Maria Quintana:** Resources, Writing—Review & Editing. **Ricardo Carvalho Cavalli:** Resources, Writing—Review & Editing. **Valerio Garrone Barauna:** Writing—Original Draft, Writing—Review & Editing, Conceptualization, Data Curation, Software, Formal Analysis, and Supervision. **Valeria Cristina Sandrim:** Writing—Review & Editing, Conceptualization, Resources, Visualization, Supervision, Project Administration, and Funding Acquisition.

## FUNDING INFORMATION

This research was supported by *Fundação de Amparo à Pesquisa do Estado de São Paulo* (FAPESP, Brazil; grant numbers 08/53593–0 [RCC], 21/12010–7, and 24/01935–8 [VCS], and 23/08897–1 [JNSK]), and by the *Conselho Nacional de Desenvolvimento Científico e Tecnológico* (CNPq, Brazil; grant numbers 308504/2021–6 and 302614/2025–7 [VCS]). VGB is supported by CNPq and by the National Institute of Science and Technology – INCT (In)Physical Activity and Health, CNPq, Brazil; grant number #408899/2024‐7.

## CONFLICT OF INTEREST STATEMENT

The authors have no conflicts of interest.

## Supporting information


**Table S1.** Analytical metrics of the three independent quality control pools.
**Table S2.** Clinical and biochemical characteristics of the study subjects at the time of diagnosis of preeclampsia.
**Table S3.** Median and mean concentrations of the 30 comparable metabolites between groups.
**Table S4.** Performance metrics for two‐metabolite predictive models.
**Table S5.** Performance metrics for three‐metabolite predictive models.
**Table S6.** Performance metrics for four‐metabolite predictive models.
**Table S7.** Performance metrics for five‐metabolite predictive models.
**Table S8.** Performance metrics for six‐metabolite predictive models.

## Data Availability

The data that supports the findings of this study are available in the supplementary material of this article.
